# PAMK Relieves LPS-Induced Enteritis and Improves Intestinal Flora Disorder in Goslings

**DOI:** 10.1155/2021/9721353

**Published:** 2021-02-22

**Authors:** Wanyan Li, Xuelian Xiang, Bingxin Li, Yifei Wang, Long Qian, Yunbo Tian, Yunmao Huang, Danning Xu, Nan Cao

**Affiliations:** ^1^College of Animal Science & Technology, Zhongkai University of Agriculture and Engineering, Guangzhou 510225, China; ^2^Guangdong Province Key Laboratory of Waterfowl Healthy Breeding, Guangzhou 510225, China

## Abstract

Polysaccharide of *Atractylodes macrocephala* Koidz (PAMK) is a biologically active component of *Atractylodes macrocephala*, which has the effect of maintaining the immune homeostasis of the body. Therefore, this study constructed a model of PAMK to relieve LPS-induced gosling enteritis and observed the morphological changes of the small intestine after HE staining. ELISA was used to detect serum CRP, IL-1*β*, IL-6, and TNF-*α* levels; immunohistochemistry was used to detect the positive rate of IgA in the small intestine; TLR4, occludin, ZO-1, cytokines, and immunoglobulin mRNA expression in the small intestine were detected by qPCR; and intestinal flora of gosling excrement was analyzed by 16S rDNA sequencing to analyze the protective effect of PAMK on goslings enteritis and the impact on intestinal flora. The results showed that PAMK relieves LPS-induced gosling enteritis by maintaining the small intestine morphology, cytokine, tight junctions, and immunoglobulin relatively stable and improving the disorder of intestinal flora.

## 1. Introduction

In the brooding stage of geese, due to the imperfection of immune function, goslings are vulnerable to various pathogenic microorganisms, leading to the occurrence of various intestinal diseases and seriously affecting the healthy development of the goslings. However, antibiotics, hormones, and other chemical drugs very easily cause drug residue in poultry products. Therefore, the development of antibiotic replacement products, which can effectively improve the immunity of goslings without residues and toxic and side effects, has become one of the hot issues of concern. Polysaccharide of *Atractylodes macrocephala* Koidz (PAMK) is the main biological activity component of *Atractylodes macrocephala*. Studies have confirmed that it has anti-inflammatory, antioxidant, antitumor, antistress, and immune-enhancing effects [1–3]. PAMK regulates immune function by promoting the development of immune organs, the proliferation and activation of immune cells, the secretion of cytokines, and the maintenance of the steady state of the immune system [4–6]. Therefore, the regulation mechanism of PAMK on gosling enteritis is worthy of further study.

The intestinal mucosal immune system is mainly composed of mucosa-associated lymphoid tissue (MALT), including mesenteric lymph nodes, aggregate lymph nodules, and lymphocytes dispersed in the intestinal lamina propria and intestinal epithelium. It plays an important role in maintaining intestinal flora balance and intestinal barrier function and repairing intestinal mucosal epithelium [7]. The strength of intestinal mucosal immune function is directly related to the structural integrity of MALT and the secretion of sIgA. SIgA mainly exists in the relative external environment of the body's digestive tract, lymphatic vessels, etc. It can prevent bacteria from attaching to the surface of the intestinal mucosa, effectively prevent bacteria from passing through the intestinal mucosa, and play an important role in strengthening the intestinal immune barrier [8–10]. The epithelial cells of the intestinal mucosa also have a barrier function, and the tight junctions of epithelial cells, such as occludin and zonula occludens-1 (ZO-1), constitute a tight junction structure. When the tight junction is closed, it can prevent the passage of bacteria or toxic macromolecules. Studies have shown that polysaccharides of traditional Chinese medicine have an important role in enhancing intestinal immunity, such as *Ganoderma atrum* polysaccharide, *Codonopsis pilosula* polysaccharide, and *Dendrobium officinale* polysaccharides have significant effects on repairing intestinal mucosal structure and enhancing intestinal mucosal immunity [10–12]. Atractylenolide I and PAMK have been shown to stimulate the migration and proliferation of intestinal epithelial cells and have a significant effect on repairing intestinal mucosal epithelium [13–15]. Studies have also reported that PAMK can enhance intestinal mucosal immune function by increasing the mRNA expression of duodenal cytokines and IgA in mice [16]. Therefore, PAMK has a significant effect on enhancing the intestinal mucosal immune function.

The intestinal microbiota is a biological barrier, forming an interdependent and interacting microecosystem with the host. The composition and ratio of various microbial species in the intestinal tract are generally relatively stable, and the bacterial species have a restrictive and interdependent relationship. The influence of intestinal flora on the immune function of intestinal mucosa is double-sided [17, 18]. When the flora is in a relatively stable state, it can form a biological barrier to prevent the displacement of bacteria and endotoxins and the colonization of exogenous pathogens. When the symbiosis of bacteria and pathogenic bacteria is imbalanced, the intestinal biological barrier function is lost, which is an important cause of intestinal diseases. Therefore, changes in intestinal flora play an important role in mucosal immune function.

Traditional Chinese medicine polysaccharides are food-borne substances that can directly enter the body's digestive system, and some of them can be directly decomposed and used as nutrients by gastrointestinal microorganisms. Therefore, traditional Chinese medicine polysaccharides must be closely related to intestinal flora. Polysaccharide of *Atractylodes macrocephala* Koidz (PAMK) is a biologically active component of *Atractylodes macrocephala*. *Atractylodes macrocephala*, belonging to Asteraceae and *Atractylodes* perennial herb, has diuretic, hypoglycemic, antitumor, antibacterial, and vasodilator effects. *Atractylodes macrocephala* is commonly used for diarrhea and other diseases and has certain effects on the treatment of gastrointestinal dysfunction [19]. Further research on the effects of *Atractylodes macrocephala* on intestinal flora found that PAMK can improve intestinal flora imbalance and have a significant effect on the treatment of colitis in mice [20]. However, the current research has not yet fully explained the effect of PAMK on the intestinal flora homeostasis. Therefore, this study built a model of PAMK to relieve LPS-induced gosling enteritis and detected immune indicators in serum and intestinal flora. Besides, 16S rDNA sequencing was performed on excrement to study the alleviation effect of PAMK on gosling enteritis and its relationship with intestinal flora.

## 2. Materials and Methods

### 2.1. Experimental Animals

One-day-old goslings (*Anser cygnoides*) were purchased from Guangdong Qingyuan Jinyufeng Goose Co., Ltd, which is a professional goose hatching company, and housed in a specific pathogen-free environment. Goslings had free access to food (including the same amount of vegetables) and water and were treated humanely, and the experiments received prior ethical approval in accordance with Zhongkai University of Agriculture and Engineering and under the approved protocol number SRM-11.

### 2.2. Reagents

PAMK (purity 70%, Lot: 20180608) was purchased from Tianyuan (Xi'an, China) and was sent to Shanghai Sanqing Biotechnology Co., Ltd. for molecular weight, monosaccharide composition, and structure analysis. GPC-RI-MALS instrument (detector: RI, MALS; mobile phase: NaNO_3_; flow rate: 0.4 mL/min; column temperature: 60°C; analytical column model: OHpak SB-805 HQ, OHpak SB-804 HQ, OHpak SB-803 HQ) was used for molecular weight determination. Weigh 10 mg of the sample, add TFA, and perform acidolysis at 110°C overnight; evaporate the mixture after acidolysis in vacuo, add 1 mL of sterile water to dissolve it thoroughly, centrifuge at 12000 rpm for 10 minutes, and take the supernatant. Using ICS (detector: DAD; mobile phase: NaOH/NaAc) to determine the composition of monosaccharides in PAMK, we found that the contents of Fuc, Gal, Glc, Xyl, and Fru were 0.98%, 0.40%, 88.67%, 4.47%, and 5.47%, respectively. In addition, the structure of PAMK was analyzed using an infrared spectrometer (VERTEX 70).

PAMK was diluted with ddH_2_O and sprayed on the feed according to the concentration of 400 mg·kg^−1^. LPS (*Escherichia coli* 055: B5, Lot: L2880) was purchased from Sigma and fully dissolved in sterile physiological saline for use. Endotoxin detection kit (chromogenic end point assay) was purchased from Fuzhou Xinbei Biochemical Industrial Co., Ltd. Enzyme-Linked Immunosorbent Assay (ELISA) kit was purchased from Nanjing Jiancheng Bioengineering Institute. TRIzol reagent (Invitrogen, China, Lot: 15596026), first-strand complementary (cDNA) kit (Invitrogen, China, Lot: K1622), and PowerUp SYBR Green Master Mix (Invitrogen, China, Lot: A25918) were used for quantitative real-time PCR analysis.

### 2.3. Treatment of Animals

Two hundred goslings (1-day-old, with half males and half females) were randomly divided into four groups (*n* = 50 per group) (see Table 1). The FBC1 and FBL groups were fed with normal diets, whereas the FBP and FBPL group were fed with 400 mg·kg^−1^ PAMK. In addition, the FBC1 and FBP groups were injected with the same amount of saline, while the FBL and FBPL groups were injected with 2 mg·kg^−1^·BW LPS per day at the 24th, 26th, and 28th day of age to construct inflammation models. Blood, excrement, duodenum, jejunum, and ileum were collected after 1 hour of injection on the 28th day. All organs were placed in liquid nitrogen immediately and stored at −80°C until used.

### 2.4. HE Staining

The duodenum, jejunum, and ileum were fixed in paraffin. The paraffin-fixed blocks were serially sectioned into 5 to 6* μ*m-thick coronal slices. For routine histological examination, the paraffin sections were stained with HE. HE-stained slices were analyzed under a Nikon fluorescence microscope (Nikon, Tokyo, Japan). The intestinal villus length and crypt depth of each group of intestines were measured using MShot Image Analysis System v1.0, and the V/C value (villus length/crypt depth) was calculated.

### 2.5. Serum Endotoxin, CRP, and Proinflammatory Factors Assay

Venous blood was collected to separate the serum. The levels of endotoxin in serum were measured using endotoxin detection kit according to the manufacturer's instruction. The levels of CRP, IL-1*β*, IL-6, and TNF-*α* in serum were measured using ELISA kit according to the manufacturer's instruction.

### 2.6. IgA Expression in the Duodenum, Jejunum, and Ileum Was Detected by Immunohistochemistry

Paraffin sections were incubated with IgA primary antibody (Bethyl, Lot: A30-103P), operated according to the immunohistochemistry kit (purchased from DAKO ChemMate EnVision, Lot: K5007), and observed using a microscope (Nikon, ECLIPSE E100). Five 400-fold magnification fields were selected for each section to calculate the number of positive IgA-secreting cells.

### 2.7. Quantitative Real-Time PCR Analysis

Specific primers matching reverse-transcribed mRNA were designed (Table 2). The procedure for quantitative PCR was similar to the one published by Yao et al. [21]. Total RNA was isolated from individual thymuses using the TRIzol reagent according to the manufacturer's instructions. First-strand complementary (cDNA) was synthesized using oligo dT primers and SuperScript II reverse transcriptase, according to the manufacturer's instructions. QPCR was performed on an ABI PRISM 7500 detection system (Applied Biosystems, USA). The PCR procedure steps included 95°C for 30 s, followed by 40 cycles of 95°C for 15 s, 60°C for 30 s, and 60°C for 30 s.

### 2.8. 16S rDNA Sequencing

The gosling excrement was collected and sent to Shenzhen Hengchuang Gene Technology Co., Ltd. for total DNA extraction, PCR amplification, and 16S rDNA sequencing. Eight replicates were set for each group. After the data analysis was performed, three samples with poor reproducibility were removed. The target DNA obtained by PCR amplification was amplified by the 16S rDNA V4 region, and the raw data was obtained by Illumina HiSeq 2500 PE250 sequencing platform. After the data was split, PE reads were spliced, tags went to the chimera sequence, etc., some low-quality data was removed and the final valid data was obtained. OTU clustering and species annotation, alpha diversity, beta diversity, and statistical analysis of differences between groups were performed on the data.

### 2.9. Statistics

The results were expressed as means ± SD. All the qPCR reactions were performed in triplicate, and the relative levels were measured in terms of threshold cycle value (Ct) and were normalized using equation 2^−∆∆Ct^. Statistical analysis of all data was performed using SPSS (version 16, SPSS Inc.). The statistical significance (*P* < 0.05) was evaluated by one-way ANOVA with simultaneous multiple comparisons among different groups.

## 3. Results

### 3.1. Infrared Spectrum Results of PAMK

**Figure 1:**
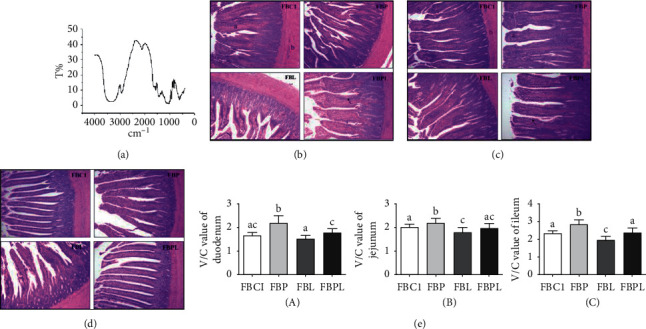
(a) Infrared spectrum results of PAMK. (b) Effects of PAMK on the morphology of duodenum in LPS-treated gosling. (c) Effects of PAMK on the morphology of jejunum in LPS-treated gosling. (d) Effects of PAMK on the morphology of ileum in LPS-treated gosling. (e) Effects of PAMK on the V/C value in LPS-treated gosling: (A) V/C value of the duodenum; (B) V/C value of the jejunum; (C) V/C value of the ileum. Letter a represents small intestine villi, b represents the muscular layer, and black arrows represent goblet cells (100×). Different letters indicate *P* < 0.05, significantly different.

Figure 1(a) shows that there are a strong and wide absorption peak near 3600 cm−1–3200 cm−1, which is O-H bond; a strong absorption peak near 2910 cm−1, which is O-H bond; and a peak around 1650 cm−1. There are a stretching vibration absorption peak, which is C=O bond; deformation vibration absorption peaks near 1450 cm−1 and 1375 cm−1, which is C-H bond; and a relatively wide absorption peak near 1100 cm−1, which is C-O bond for pyran ring structure.

### 3.2. PAMK Protects Intestinal Morphology from LPS in Goslings

The results in Figure 1(b) show that the villi of the FBC1 and FBP are compact, the crypts are deep, the tissues are dense, and the intestinal structure is intact. In the FBL, the villi were severely broken and the defect was severe. The number of cells in the villus epithelial layer was reduced, the cells were vacuolated, and the connective tissue in the submucosa was loose. Compared with the FBL, the FBPL group's tissue structure was significantly reduced. The number of villous epithelial cells was similar to that of the FBC1 group. The width and number of villi were significantly increased. The overall morphology was close to the FBC1 group, but the lymphocyte infiltration was indistinguishable from the FBL group. Therefore, after feeding with PAMK, it has obvious repairing effect on LPS-induced injury, which can make the morphology and quantity of villi close to FBC1 and reduce the number of goblet cells in intestinal crypt.

Figure 1(c) shows that the villi of the FBC1 and FBP groups are neatly arranged, and the number of goblet cells in the villi and crypt is significantly larger than that in the duodenum. In the FBL group, the epithelial detachment of jejunum villi was severe, the number of intestinal glands increased, and the number of lymphocytes was more. The number of goblet cells in the villus epithelium was significantly higher than that in the FBC1 group. The jejunum morphology of the FBPL group was close to that of the FBC1 group. The villi were neatly arranged. The goblet cells in the villus epithelium were significantly higher than those in the FBC1 group. The tissue compaction recovery was better, the gut gland structure was clear, the thickness was normal, the congestion was reduced, and the lymphatic infiltration phenomenon and FBL were observed. The groups are similar. In summary, PAMK has no obvious effect on the structure of jejunum, but it also has a good ability to repair the jejunum under LPS injury, which can maintain the integrity of intestinal villi and increase the length of villi.

As shown in Figure 1(d), compared with the FBC1 group, the ileal villi of the FBP group were broadened, and the number of goblet cells in the villus epithelial area increased, evenly distributed between the aligned columnar cells. In the FBL group, the villi became thicker and shorter, the gap increased, the epithelial detachment of the villus was severe, the vacuolar cells increased, the number of the goblet cells increased, the cells in the lamina propria was increased, and the number of lymphocytes increased. Lymphocyte infiltration and hyperemia are evident, and the toroidal muscle layer becomes thinner. In the FBPL, the length and width of the ileum were close to those of the FBC1 group, but there was still epithelial shedding at the top of the villus. In addition, the number of goblet cells in the villi was close to that of the FBC1 group, and the lamina propria structure returned to normal. The thickness of the gut was also close to that of the FBC1 group. In summary, PAMK has a significant effect on the development of ileal villi in normal goslings, and this effect only occurs in the ileum. In addition, PAMK has a significant repair effect on the damage caused by LPS. Except for the epithelial detachment at the top of the villus, the other lesions can be relieved obviously, and the villus morphology returns to normal.

By calculating the villus length/crypt depth (V/C) values of the duodenum, jejunum, and ileum (Figure 1(e)), it was found that the V/C values of the FBL group in the jejunum and ileum were significantly lower than those in the FBC1 group (*P* < 0.05), indicating that LPS can cause a decrease in the V/C value. In addition, the V/C values of the FBP in the duodenum, jejunum, and ileum were significantly higher than those in the FBC1 group (*P* < 0.05), indicating that PAMK can increase the V/C value of the small intestine and promote the development of small intestine villi. At the same time, the V/C values of the FBPL group in the duodenum and ileum were significantly higher than those in the FBL (*P* < 0.05), indicating that PAMK has protective and repairing effects on the duodenal and ileal mucosa of LPS injury.

### 3.3. PAMK Alleviates Intestinal Inflammation Induced by LPS in Goslings

**Figure 2:**
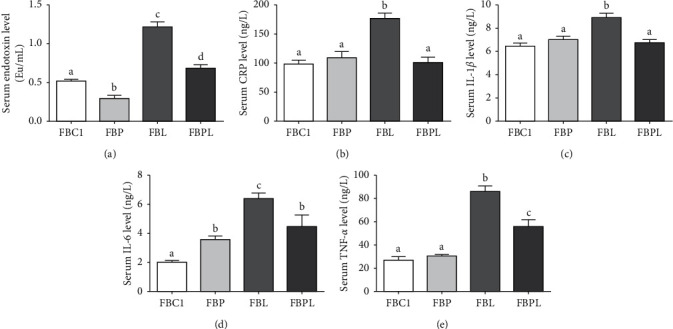
Effects of PAMK on the serum inflammation index of LPS-treated gosling. (a) The serum endotoxin level. (b) The serum CRP level. (c) The serum IL-1*β* level. (d) The serum IL-6 level. (e) The serum TNF-*α* level (*n* = 50). Different letters indicate *P* < 0.05, significantly different.

The results showed that (Figure 2) the levels of endotoxin, CRP, and proinflammatory factors (IL-1*β*, IL-6, TNF-*α*) in the serum of FBL group were significantly increased compared with FBC1 group (*P* < 0.05). It means that LPS significantly enhanced the inflammation of goslings and successfully constructed an LPS gosling inflammation model. Compared with the FBC1, the serum levels of endotoxin in the goslings of the FBP were significantly decreased, and IL-6 was significantly increased (*P* < 0.05), while CRP, IL-1*β*, and TNF-*α* were not significantly changed (*P* > 0.05). However, the levels of endotoxin, CRP, and proinflammatory factors (IL-1*β*, IL-6, TNF-*α*) in the FBPL were significantly lower than those in the FBL (*P* < 0.05). Among them, CRP and IL-1*β* could be restored to the level of FBC1, while the levels of endotoxin, IL-6, and TNF-*α* were still significantly higher than those of FBC1 group (*P* < 0.05). The results showed that PAMK can not only reduce the endotoxin in healthy goslings, but also protect the goslings from LPS, reducing the inflammation in the gosling induced by LPS.

**Figure 3:**
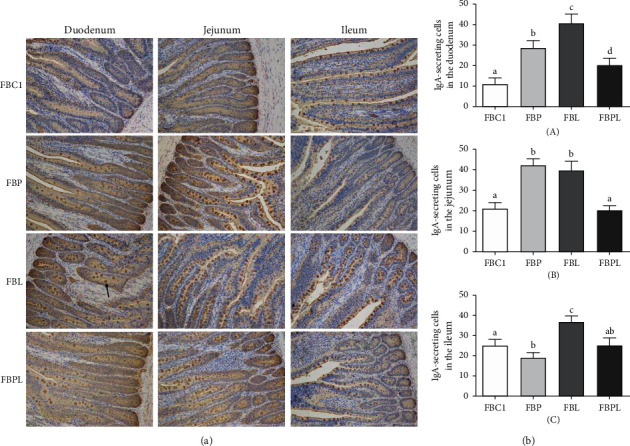
(a) Effects of PAMK on IgA-secreting cells in small intestine of LPS-treated gosling. Black arrows indicate IgA-secreting cells (200×). (b) Effects of PAMK on number of IgA-secreting cells in small intestine of LPS-treated gosling: (A) number of IgA-secreting cells in the duodenum; (B) number of IgA-secreting cells in the jejunum; (C) number of IgA-secreting cells in the ileum. Different letters indicate *P* < 0.05, significantly different.

The results of duodenum immunohistochemistry showed (Figures 3(a) and 3(b) (A)) that there were no obvious IgA-secreting cells in the intestinal epithelial cells and crypts of the FBC1 group, indicating that the sIgA secretion ability of the duodenum was weak. Compared with FBC1 group, IgA-secreting cells in FBP group increased significantly (*P* < 0.05), mainly distributed in intestinal villus epithelial cells, indicating that PAMK can promote the increase of IgA-secreting cells in the villus epithelium of duodenum, and the secretion of sIgA is enhanced. In addition, the number of IgA-secreting cells in the duodenal villi of the FBL group was significantly higher than that of the FBC1 group and the FBP group (*P* < 0.05), and the number of IgA-secreting cells was the highest among the four groups, indicating that IgA cells were significantly increased and LPS significantly promoted the secretion capacity of sIgA. However, the number of IgA-secreting cells in the FBPL group was significantly lower than that in the FBL group (*P* < 0.05), and the IgA-secreting cells in the crypt were less than those in the FBL group, indicating that PAMK can alleviate the IgA-secreting cells compensatory increase caused by LPS in the duodenum.

The results of jejunal immunohistochemistry showed (Figures 3(a) and 3(b) (B)) that the jejunum of the four groups of goslings contained more IgA-secreting cells. Compared with FBC1, the number of IgA-secreting cells in the jejunum of FBP group and FBL group was significantly increased (*P* < 0.05), the staining was deeper, and the cell morphology and contour were more obvious. The number of IgA-secreting cells in the jejunum of the FBPL group was not significantly different from that of the FBC1 group (*P* > 0.05), and the morphology of IgA-secreting cells was more similar to that of the FBC1 group. Besides, both PAMK and LPS could significantly increase (*P* < 0.05) the number of jejunal IgA-secreting cells and promote the secretion of sIgA. After cotreatment with PAMK and LPS, the number of jejunal IgA-secreting cells was close to that of FBC1 group.

The results of ileal immunohistochemical staining showed (Figures 3(a) and 3(b) (C)) that the IgA-secreting cells in FBC1 group were mainly distributed in intestinal villus epithelial cells, and the IgA-secreting cells in FBP group were significantly decreased compared with FBC1 group (*P* < 0.05). Compared with the FBC1 group, the IgA-secreting cells of the FBL group were significantly increased (*P* < 0.05) and mainly distributed in the intestinal mucosa epithelium and crypt. The IgA-secreting cells in the FBPL group were significantly lower than those in the FBL group (*P* < 0.05), and there was no significant difference between the FBP group and the FBC1 group (*P* > 0.05). The results showed that PAMK reduced the number of IgA-secreting cells in the ileum of healthy goslings and also decreased the increase of IgA-secreting cells in the ileum caused by LPS.

In summary, LPS caused an increase in IgA-secreting cells in the duodenum, jejunum, and ileum, promoted small intestine to secrete sIgA, and enhanced the immune barrier function of intestinal mucosal immunity. However, whether this is due to the compensatory increase of IgA secretory cells caused by the inflammation of LPS still needs further discussion. In addition, PAMK has the effect of increasing the number of IgA-secreting cells in the duodenum and jejunum and also inhibits the significant increase in the number of IgA-secreting cells in the small intestine caused by LPS. Therefore, PAMK is a dynamic and stable regulation of intestinal mucosal immune function.

**Figure 4:**
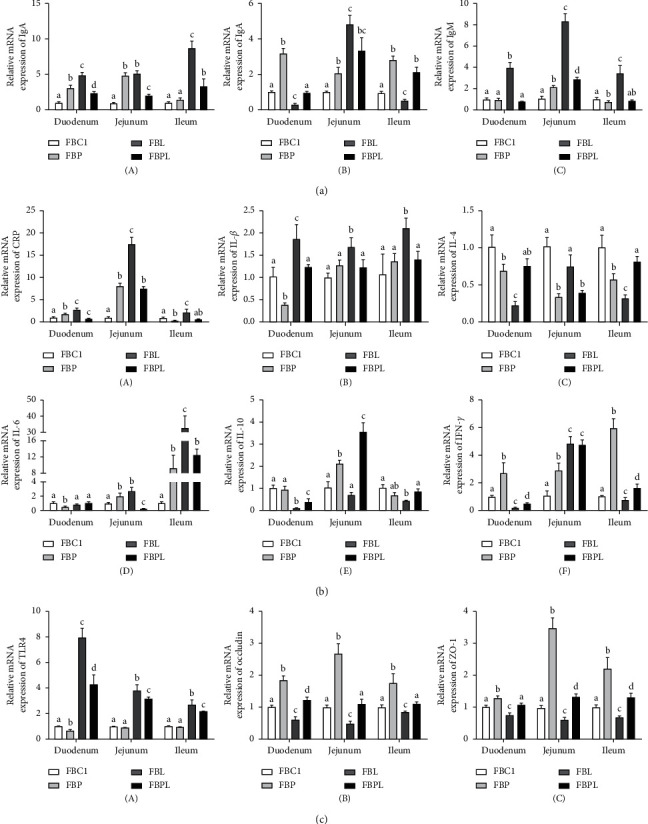
(a) Effects of PAMK on the immunoglobulin mRNA expression in the small intestine of LPS-treated gosling: (A) relative mRNA expression of IgA; (B) relative mRNA expression of IgG; (C) relative mRNA expression of IgM. (b) Effects of PAMK on the cytokine mRNA expression in the small intestine of LPS-treated gosling: (A) relative mRNA expression of CRP; (B) relative mRNA expression of IL-1*β*; (C) relative mRNA expression of IL-4; (D) relative mRNA expression of IL-6; (E) relative mRNA expression of IL-10; (F) relative mRNA expression of IFN-*γ*. (c) Effects of PAMK on the small intestine TLR4 and tight junction mRNA expression of LPS-treated gosling: (A) relative mRNA expression of TLR4; (B) relative mRNA expression of occludin; (C) relative mRNA expression of ZO-1 (*n* = 6). Different letters indicate *P* < 0.05, significantly different.

The relative mRNA expression of IgA in the gosling small intestine showed (Figure 4(a) (A)) that the IgA mRNA expression in the duodenum, jejunum, and ileum of FBL was significantly (*P* < 0.05) higher than that of FBC1, indicating that LPS would significantly increase the expression of IgA mRNA in the small intestine. In addition, the relative mRNA expression of IgA in duodenum and jejunum of FBP was significantly (*P* < 0.05) higher than that of FBC1, but in duodenum and ileum it was significantly (*P* < 0.05) lower than that of FBL. At the same time, relative mRNA expression of IgA in duodenum, jejunum, and ileum of FBPL was significantly (*P* < 0.05) lower than that of FBL. This shows that PAMK can promote the expression of IgA mRNA in the small intestine of healthy goslings, but it can also alleviate the excessive increase of IgA mRNA in the small intestine of goslings treated by LPS and make the expression of IgA closer to that of healthy goslings.

The relative mRNA expression of IgG in the goslings small intestine tissue showed (Figure 4(a) (B)) that the IgG mRNA expression in the jejunum of FBL was significantly (*P* < 0.05) higher than that of FBC1, but in duodenum and ileum it was significantly (*P* < 0.05) lower. This shows that LPS can significantly change the IgG mRNA expression in the small intestine. In addition, FBP duodenum, jejunum, and ileum IgG mRNA expression were significantly (*P* < 0.05) higher than that of FBC1, and the expression in duodenum and ileum of FBPL was significantly (*P* < 0.05) higher than that of FBL. This shows that PAMK can promote the expression of IgG mRNA in the small intestine of healthy goslings, but it can also alleviate the change of IgG mRNA expression in the small intestine of goslings treated by LPS, so that the expression of IgA is closer to that of healthy goslings.

The relative mRNA expression of IgM in the small intestine tissue of goslings showed (Figure 4(a) (C)) that the mRNA expression of IgM in duodenum, jejunum, and ileum of FBL was significantly (*P* < 0.05) higher than that of FBC1, indicating that LPS would significantly (*P* < 0.05) increase the mRNA expression of IgM in the small intestine. In addition, the mRNA expression of IgM in the jejunum of FBP was significantly (*P* < 0.05) higher than that of FBC1, but in the ileum it was significantly (*P* < 0.05) lower than that of FBC1. At the same time, mRNA expression of IgM in duodenum, jejunum, and ileum of FBPL was significantly (*P* < 0.05) lower than that of FBL. This shows that PAMK can alleviate the excessive increase of IgM mRNA expression in the small intestine of goslings treated with LPS and make the expression of IgM closer to that of healthy goslings.

The relative mRNA expression of CRP in the small intestine tissues of goslings showed (Figure 4(b) (A)) that the expression of CRP mRNA of FBL in duodenum, jejunum, and ileum was significantly (*P* < 0.05) higher than that of FBC1, indicating that LPS increased the expression of CRP mRNA in the small intestine and promoted inflammation happened. In addition, the CRP mRNA expression in duodenum and jejunum of FBP was significantly (*P* < 0.05) higher than that of FBC1, while it was significantly (*P* < 0.05) lower than that of FBC1. However, the expression of CRP mRNA in duodenum, jejunum, and ileum of FBPL was significantly (*P* < 0.05) lower than that of FBL. This shows that PAMK can alleviate the significant increase of CRP mRNA expression in the small intestine of goslings treated with LPS, so that the expression of CRP is closer to that of healthy goslings.

The relative mRNA expression of IL-1*β* in the small intestine tissues of goslings showed (Figure 4(b) (B)) that the mRNA expression of IL-1*β* of FBL in duodenum, jejunum, and ileum was significantly (*P* < 0.05) higher than that of FBC1. However, the mRNA expression of FBPL in duodenum, jejunum, and ileum had no significant (*P* > 0.05) difference from that of FBC1. This shows that PAMK can alleviate the significant increase of IL-1*β* mRNA expression in the small intestine of goslings treated with LPS, so that the expression of IL-1*β* is closer to that of healthy goslings.

The relative mRNA expression of IL-4 in the small intestine tissues of goslings showed (Figure 4(b) (C)) that the mRNA expression of IL-4 of FBL in duodenum and ileum was significantly (*P* < 0.05) lower than that of other groups. However, expression in FBPL was significantly (*P* < 0.05) higher than that in FBL and had no significant (*P* > 0.05) difference from that in FBC1. Besides, mRNA expression of IL-4 of FBP and FBPL in jejunum was significantly lower than that of FBC1.

The relative mRNA expression of IL-6 in the small intestine tissues of goslings showed (Figure 4(b) (D)) that the mRNA expression of IL-6 of FBL in jejunum and ileum was significantly (*P* < 0.05) higher than that of FBC1. However, expression in FBPL was significantly (*P* < 0.05) lower than that in FBL. The mRNA expression of IL-6 of FBP in duodenum was significantly (*P* < 0.05) lower than that of FBC1 and had no significant (*P* > 0.05) difference from other groups.

The relative mRNA expression of IL-10 in the small intestine tissues of goslings showed (Figure 4(b) (E)) that the mRNA expression of IL-10 of FBL in duodenum and ileum was significantly (*P* < 0.05) lower than that of FBC1. However, expression in FBPL was significantly (*P* < 0.05) higher than that in FBL. Besides, the mRNA expression of IL-10 of FBP and FBPL in jejunum was significantly (*P* < 0.05) higher than that of the other two groups.

The relative mRNA expression of IFN-*γ* in the small intestine tissues of goslings showed (Figure 4(b) (F)) that the mRNA expression of IFN-*γ* of FBL in duodenum and ileum was significantly (*P* < 0.05) lower than that of FBC1. However, expression in FBPL was significantly (*P* < 0.05) higher than that in FBL. Besides, the mRNA expression of IFN-*γ* of FBL and FBPL in jejunum was significantly (*P* < 0.05) higher than that of the other two groups.

The results showed (Figure 4(c) (A)) that the mRNA expression levels of TLR4 in the duodenum, jejunum, and ileum of the FBL group were significantly higher than those in the FBC1 group (*P* < 0.05), indicating that LPS upregulated the expression of TLR4 in the small intestine, and the LPS model was built successfully. Compared with FBC1, the mRNA expression of TLR4 in the duodenum of FBP group was significantly decreased (*P* > 0.05), but there was no significant change in jejunum and ileum. However, the mRNA expression of TLR4 in the duodenum, jejunum, and ileum of the FBPL group was significantly decreased (*P* < 0.05) but could not be reduced to the level of FBC1. The results indicated that PAMK can inhibit the TLR4 inflammatory signaling pathway in LPS-treated goslings and has a certain anti-inflammatory effect.

The results showed (Figures 4(c) (B) and 4(c) (C)) that the mRNA expression levels of occludin and ZO-1 in the duodenum, jejunum, and ileum of the FBL group were significantly lower than those in the FBC1 group (*P* < 0.05). This indicated that LPS damaged the mucosal barrier function of the small intestine of goslings. Compared with the FBC1 group, the mRNA expression levels of occludin and ZO-1 in the duodenum, jejunum, and ileum of the FBP group were significantly increased (*P* < 0.05). The mRNA expression levels of occludin and ZO-1 in goslings of FBPL group returned to normal levels, indicating that PAMK can alleviate the damage of LPS on the intestinal mucosal barrier of goslings. In conclusion, PAMK can promote the mRNA expression of tight junction occludin and ZO-1 in the small intestine of healthy and enteric inflammatory goslings, indicating that PAMK has a protective effect on the intestinal tight junction of the small intestine.

### 3.4. PAMK Improves LPS-Induced Gut Microbial Disorders in Gosling

**Figure 5:**
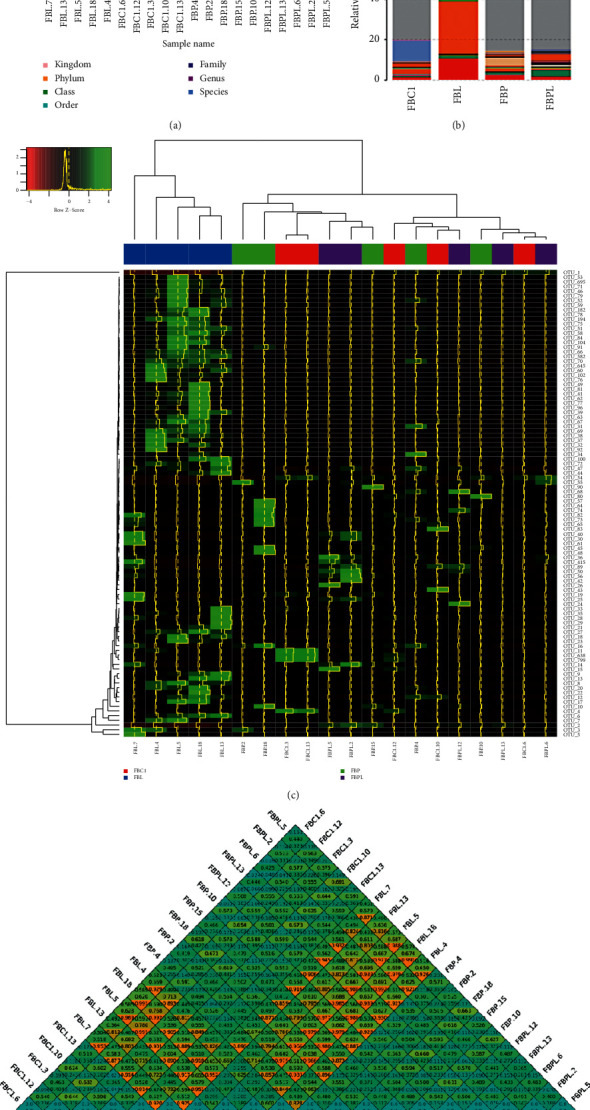
(a) The sequence composition of samples at each taxonomic level. Sequence number percent indicates the ratio of the number of sequences annotated to this level to the total annotation data. (b) Annotation results of grouping relative abundance (species). Relative abundance is the ratio of the number of bacteria that are annotated to species to the total number of bacteria. (c) Species abundance cluster. (d) Heatmap of beta diversity index. The number in the square is the coefficient of dissimilarity between two pairs of samples. The smaller the coefficient of difference is, the smaller the difference of species diversity is. In the same box, the values of upper, middle, and lower represent weighted UniFrac, unweighted UniFrac, and Bray–Curtis distance.

As shown in Figure 5(a), there is no significant difference in the sequence composition ratio of each sample at the boundaries, gates, classes, orders, and families. There are differences between groups at the level of genus and species. The results showed that the sequence composition of the FBL decreased at the genus level, and the proportion at the species level increased. The sequence composition ratio of the other three groups was similar at the genus and species level, and there was no significant change.

Based on the species annotation and abundance information of all samples at the genus level, the abundance information of the genus level in each sample was selected to draw a histogram, and the difference in abundance levels between the groups was analyzed. The results (Figure 5(b)) showed that the species abundance of the FBC1, FBP, and FBPL group was lower at the genus level. However, the bacterial classification of the FBL group was significantly different from that of the other three groups, and the species abundance was significantly higher than the other three groups.

In order to find the aggregation law of species or samples, the abundance information of the genus level in each sample was selected to draw a heatmap and clustered from the classification information and the difference between the samples. The results (Figure 5(c)) show that the samples in the FBL group are clustered together, indicating that the sample abundance of the group is very similar and the repeatability is good. In addition, samples from the FBL group had high *Z* values in many annotated species classifications, indicating a higher species abundance in the LPS group. However, the clustering results of the other three groups did not show differences, indicating that the abundances of the three groups were very similar and the abundance was low. From the above results, LPS treatment can significantly increase the species abundance of gosling gut microbes, while adding 400 mg/kg PAMK can maintain the relative stability of microbial species abundance in the gut of goslings, making it closer to healthy goslings.

**Figure 6:**
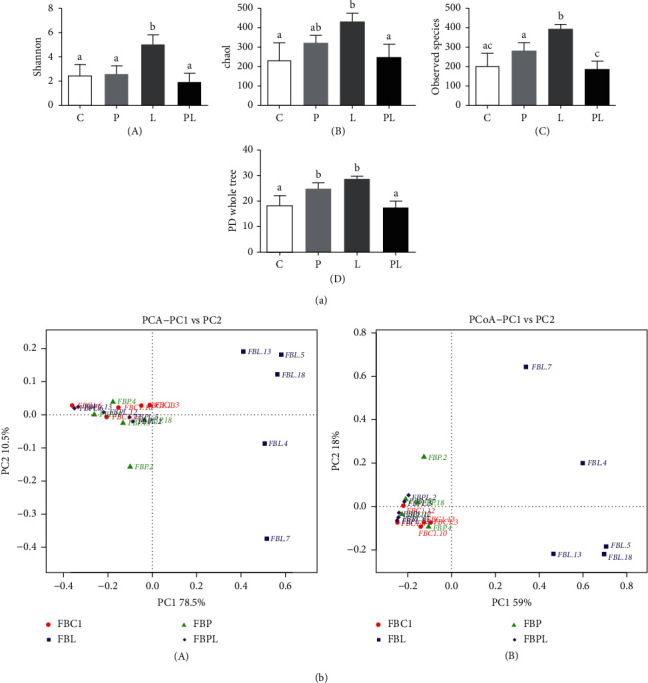
(a) Alpha diversity index: (A) Shannon; (B) Chao1; (C) observed species; (D) PD whole tree. Different letters indicate *P* < 0.05, significantly different. (b) Results of PCA and PCoA: (A) PCA plot based on the genus level; (B) PCoA plot based on the Bray–Curtis distance.

Alpha diversity is an analysis of the richness and uniformity of species composition in a sample, usually assessed using indicators such as observed species, PD whole tree, Shannon, and Chao1. The more complex the diversity of a sample, the higher the index. The results showed (Figure 6(a)) that the Shannon, Chao1, observed species, and PD whole tree indices in the FBL group were the highest among the four groups and were significantly different from those in the FBC1 and FBPL groups (*P* < 0.05). This means that the diversity of microbial species in the gut of the FBL group is very complicated, and the addition of 400 mg/kg PAMK can maintain the stability of microbial species diversity in the intestines of the goslings.

Beta diversity was used to compare the microbial community composition between different samples. Bray–Curtis, weighted UniFrac, and unweighted UniFrac distance were used to estimate the microbial community structure differences between different samples based on the OTU abundance information of the samples. Beta diversity is mainly used to measure the coefficient of dissimilarity between two samples. The smaller the value, the smaller the difference in microbial community composition between the two samples.  Figure 5(d) is a beta diversity index heatmap based on the weighted UniFrac distance, Bray–Curtis distance, and unweighted UniFrac distance for each sample. The results showed that the individual coefficients of each sample in the FBC1, FBP, and FBPL groups were relatively small, indicating that the species diversity between these samples was small. However, the differences in the FBL group and the other three groups were larger, indicating that the species diversity between the FBL group and the other three groups was large.

Principal Component Analysis (PCA) is a method of dimensionality reduction of multidimensional data to extract the most important elements and structures in the data. Principal Coordinate Analysis (PCoA) is a similar dimension reduction method to PCA, extracting the most important elements and structures from multidimensional data. The results show (Figure 6(b) (A)) that the first principal component of PCA accounts for 78.5% and the second principal component accounts for 10.5%, while the first principal component of PCoA results is 59% and the second principal component accounts for 18% (Figure 6(b) (B)). In addition, both results showed that all samples in the FBC1 group, FBP group, and FBPL group were close to each other in the first principal component and farther away from each sample in the FBL group. This indicated that the species composition of FBC1 group, FBP group, and FBPL group was similar, while the species composition structure of FBL group was far from that of the other groups. The results showed that PAMK can maintain the stability of the gut microbial species composition and prevent the imbalance of intestinal microbial species composition caused by LPS.

The ANOSIM (analysis of similarities) is a nonparametric test used to test whether the difference between the groups is significantly greater than the difference within the group to determine whether the test is meaningful. The results of the ANOSIM mainly include the *R* value and the *P* value between the groups. The *R* value is within (−1, 1). The *R* value is greater than 0, indicating that the difference between the groups is significant. The *R* value is less than 0, indicating that the difference within the group is greater than the difference between the groups. The reliability of the statistical analysis was expressed by the *P* value, and *P* < 0.05 indicates that the statistics were significant. From the results in Table 3, there was a significant difference between the FBL group and the other three groups, but there was no significant difference between the FBC1 group, the FBP group, and the FBPL group.

Based on Fisher's exact test MetaStat software, the statistical analysis of species differences was performed. The *P* value of the multiple-comparison test was corrected by FDR to obtain the *Q* value. Finally, the species with significant differences between different groups were selected according to *P* value and *Q* value. As can be seen from Table 4, at the genus level, the abundance of *Romboutsia* in the FBL group was significantly lower than that in the other three groups (*P* < 0.05;  *Q* < 0.05), while there were no significant differences between the FBC1, FBP, and FBPL group. The results showed that LPS caused a decrease in *Romboutsia* in the intestines of goslings, while the abundance of *Romboutsia* in the FBPL group was close to that of healthy goslings and remained relatively stable.

## 4. Discussion

LPS is the main component of the cell wall of Gram-negative bacteria and plays an important role in mediating the intestinal inflammatory response of inflammatory bowel disease, so it is often used to construct enteritis models for more in-depth scientific research. Research points out that the mechanism of LPS-induced damage to the body has the following two types [22–24]. The first is to act on target cells such as immune cells and epithelial cells to make surface molecules abnormal and increase cell permeability, resulting in functional damage. The second is by activating immune cells to secrete large amounts of inflammatory mediators and cause inflammation. The experiments in this paper found that the duodenum, jejunum, and ileum tissue damage caused by LPS of goslings was obvious; the levels of endotoxin, CRP, and proinflammatory factors (IL-1*β*, IL-6, TNF-*α*) in serum were significantly increased; the number of IgA-secreting cells increased dramatically; the expression of TLR4 increased; and the expression of tight junction occludin and ZO-1 decreased significantly. In addition, LPS caused an imbalance in intestinal microbial species composition, species abundance, and diversity. Combining the changes of the above indicators, we find that that goslings have enteritis; that is, the LPS-induced enteritis model is successfully constructed.

The intestine not only has the functions of digestion, absorption, and secretion, but also has an important barrier function and is the main part of the body's mucosal immune system. The mucosal barrier of the body includes mechanical barrier, chemical barrier, immune barrier, and biological barrier [25]. Mechanical barrier refers to the tight connection between intestinal mucosa epithelium and epithelial cells with good morphological structure. Chemical barrier refers to mucus and bacteriostatic substances in the intestinal cavity. Immune barrier refers to sIgA in intestinal mucosa lymphoid tissue and intestinal cavity. The biological barrier refers to the intestinal probiotics that have the function of resisting harmful foreign bacteria. The mechanical barrier, chemical barrier, immune barrier, and biological barrier do not operate independently but coordinate and cooperate with each other to jointly maintain the normal intestinal barrier function of the intestine, so that the entire intestinal mucosal immunity is in a healthy and stable response state [26]. This study found that PAMK increases the ratio of intestinal V/C ratio, that is, increases the surface area of intestinal villi and increases the absorption function of goslings, indicating that PAMK can promote the development of small intestine villi. In addition, PAMK can alleviate the decrease in the V/C ratio caused by LPS, indicating that PAMK has a protective effect on the development and morphological structure of the small intestinal mucosa.

The normality of the morphological structure of the small intestine mucosa is directly related to the function of the intestinal barrier and reflects the level of intestinal mucosal immunity. Observing the small intestine of goslings in this study, we found that LPS has obvious damage to the small intestine of healthy goslings. The overall performance is the loss of villi epithelial damage, cellular vacuolation, increased compensation for goblet cells, loose tissue, and obvious inflammation. Goslings show the characteristics of enteritis, and the mechanical barrier of the small intestine is damaged. We found that LPS has the most significant damage to the duodenum and less damage to the jejunum segment, which may be directly related to the high expression of TLR4 in the duodenal tissue of goslings. PAMK has a good repair effect on the damaged small intestine mucosa, of which the repair of the ileum segment is the most significant, and can promote the development of the ileum villi of healthy goslings. Therefore, it can be concluded that PAMK has a certain promoting effect on the development of the small intestine. It can effectively alleviate tissue inflammation and mechanical barrier damage caused by LPS. This effect is most obvious in the ileal segment, and the mechanism of action can be further explored. Intestinal epithelial cells can maintain the permeability of small intestinal epithelium by controlling the opening and closing of tight junctions. Therefore, tight junctions have an important role in preventing pathogenic bacteria and harmful antigens from entering the intestinal mucosa lamina propria and are the structural basis for maintaining the mechanical barrier function of the intestinal epithelium [26, 27]. LPS, at physiological concentrations, causes an increase in intestinal tight junction permeability (TJP) via TLR4-dependent activation of the FAK/MyD88/IL-1R-associated kinase 4 signaling pathway [28]. Further research found that LPS downregulated the expression of occludin and ZO-1 mRNAs, but *Astragalus* polysaccharide reduces inflammatory response by upregulating the mRNA expression of ZO-1 and occludin and decreasing permeability of LPS-infected Caco-2 cells [29, 30].

This study found that PAMK can not only promote the expression of occludin and ZO-1 in the duodenum, jejunum, and ileum of goslings, but also alleviate the inhibitory effect of LPS on occludin and ZO-1, so that the expression of occludin and ZO-1 in each small intestine segment is close to or higher than that of normal goslings, which has a protective effect on the tight connection of goslings. In summary, PAMK can alleviate the inflammatory response of LPS, maintain the normal intestinal mechanical barrier function, and ultimately protect the immune function of goose intestinal mucosa. The report pointed out that PAMK can cause the increase of cellular polyamine content, stimulate the migration of intestinal epithelial cells, and accelerate intestinal injury and healing, which may be the mechanism of PAMK protection and repair of mechanical barriers [14]. In addition, the effect of PAMK on the expression of occludin and ZO-1 was most obvious in the jejunum segment, while the protective effect of PAMK on the morphology of the jejunum segment was the least. We speculate that PAMK protects the goose intestinal mechanical barrier through the coordination and complementation of intestinal morphology and tight connection. When the tissue structure of a certain small intestine is damaged, the body can enhance the function of tight junctions to maintain a mechanical barrier, but the specific mechanism of action still needs more in-depth research.

Plasma cells differentiated from intestinal B lymphocytes undergo a process of polymorphism change and class conversion at the center of intestinal lymphocytic vesicles and then convert Ig type cells into mucosa-related IgA-secreting cells. Therefore, the content of sIgA in the intestine is directly related to the number and secretion capacity of IgA-secreting cells, and its secretion is also regulated by the level of receptor humoral immunity. Intestinal sIgA is an important marker of intestinal acquired immunity, which can prevent bacteria from adhering to the surface of intestinal epithelial cells, neutralize toxins, and viruses and have an immunoprotective effect [25]. Therefore, the maintenance of intestinal homeostasis depends largely on the role of sIgA [31]. The current research proves that the traditional Chinese medicine polysaccharides such as *Astragalus* polysaccharide, epimedium polysaccharide, and PAMK can promote intestinal secretion of sIgA [32]. This study also found that PAMK can increase the number of IgA-secreting cells in the duodenum and jejunum of healthy goslings. It is speculated that PAMK mainly increases the secretion of sIgA in the duodenum and jejunum. The secretion of SIgA is related to intestinal epithelial and lamina propria immune cells. These immune cells contain antigen recognition receptors, TLRs. By recognizing these receptors, they can mediate B cell differentiation and eventually produce noninflammatory immunoglobulin sIgA. Therefore, the effect of *Atractylodes macrocephala* polysaccharides on the number of IgA-secreting cells in different segments of the small intestine is different, which may be related to the inconsistent expression of TLR receptors in various intestinal segments. In addition, after LPS treatment, sIgA secretion in goose duodenum, jejunum, and ileum was the highest among the four treatment groups. This study also found that, after LPS treatment, the expression of TLR4 in the duodenum, jejunum, and ileum of goslings was also the highest among the four groups, and the order of TLR4 expression from high to low was duodenum, jejunum, and ileum. Therefore, we explain that the increase in IgA-secreting cells after LPS treatment indirectly leads to an increase in sIgA secretion, which is related to the expression level of TLR4 receptor. LPS as a ligand, after binding to TLR4, mainly causes the activation of downstream inflammatory pathways. Therefore, it is speculated that the increase of IgA secretory cells in goslings caused by LPS is a result of inflammatory response, and PAMK has the effect of reducing the number of IgA secretory cells. This study also found that PAMK can reduce the endotoxin content in the serum of healthy goslings and also make the inflammation indicators (endotoxin, CRP, IL-1*β*, IL-6, TNF-*α*) lower. In summary, PAMK reduces the number of IgA-secreting cells in various segments of gosling intestine and inflammation indicators in serum, indicating that PAMK has a mitigating effect on the inflammation caused by LPS and has a significant role in maintaining the intestinal immune barrier function.

The stability of the biological barrier function is not the absolute increase or decrease of the bacterial flora, but the mutual inhibition of different bacterial flora to achieve the relative balance of various intestinal bacteria, thereby effectively maintaining the microecological balance of the intestine and promoting the mucosal immune system stable. The two most important ways for polysaccharides of traditional Chinese medicine (TCM) to play a role are as follows: first, polysaccharides can be directly absorbed by the intestine into the blood circulation without being degraded or degraded; second, the sugar fragments produced by the partial degradation of sugar by the bacteria directly act on the epithelial cells [33]. Therefore, many TCM polysaccharides have been proven to maintain or improve the composition of intestinal flora. For example, *Polygonatum sibiricum* polysaccharides can prevent type 2 diabetes by regulating intestinal microbes in rats [34]; *Ganoderma lucidum* polysaccharides can regulate obesity-related metabolic disorders by improving intestinal microflora imbalance [35]; and PAMK can significantly improve the intestinal microflora disorder [36]. The results of this study also show that PAMK can significantly improve the imbalance of the intestinal flora of goslings caused by LPS. LPS causes a significant reduction in the flora of the genus *Romboutsia*, and PAMK mainly improves the stability of the abundance of the flora of the genus *Romboutsia*, thereby effectively improving the imbalance of intestinal flora. In 2014, scientists first isolated and identified a new type of bacterium *Romboutsia* in the human colon and officially created a new genus *Romboutsia* that year [37]. *Romboutsia* is a Gram-positive bacterium, which belongs to anaerobic bacteria in the intestine and belongs to *Streptococcus*, which can form spores [38]. Reports showed that the dicaffeoylquinic acid of Kudingcha as a dietary polyphenol is a new type of prebiotics, which has a significant regulating effect on intestinal microflora and significantly increases the abundance of *Romboutsia* [39]. Besides, Chen et al. detected the dynamics of the cecal microbial community in AA broilers challenged with *Eimeria tenella* and found that microbial shifts occur during the infection and *Romboutsia* decreased in abundance [40]. This shows that *Romboutsia* is a probiotic in the intestine, and increasing its abundance is beneficial to the body. The present study indicates that oral administration of YM001 altered the diversity and composition of intestinal microbiota in tilapia, such as *Romboutsia*, but these changes were only temporary, nonlethal, and recoverable [41]. Therefore, the effect of PAMK on the stability of the flora may be accomplished by regulating the abundance of *Romboutsia*. However, the specific mode of action of PAMK to adjust the abundance of *Romboutsia*, the mutual adjustment relationship between *Romboutsia* and other flora, and what common diseases *Romboutsia* is related to need further research.

## 5. Conclusions

The study found that PAMK relieves LPS-induced gosling enteritis by maintaining the small intestine morphology, cytokine, tight junctions, and immunoglobulin relatively stable and improving the disorder of intestinal flora.

## Figures and Tables

**Table 1:** Experiment grouping and treatment.

Group	PAMK (mg/kg)	LPS (mg/kg·BW)
FBC1	—	—
FBP	400	—
FBL	—	2
FBPL	400	2

**Table 2:** The primers and amplification parameters of target genes.

Gene	Primer sequence (5′ to 3′)	
TLR4	F: TTGTGTGCTGAAGGTCCAAG	R: TCCTCAGTTTCCTGGGTCTG
Occludin	F: TCTTCCACATCAAGCGCATG	R: GGTCGCTTTGGATGTTGGTT
ZO-1	F: CAAAGGTGAAGTGTTCCGGG	R: CTCCTCCTGCTGTCTTTGGA
IL-1*β*	F: ACGGTGTGGGGACATTCATC	R: AGGCGAAGCTTCTTCTGTGG
IL-2	F: TCATCTCGAGCTCTACACACCAA	R: TGCATTCACTTCCGGTGTGAT
IL-4	F: GGCATCTACCTCAACTTGCT	R: CTCTTTCGCTACTCGTTGGA
IL-6	F: ACGATAAGGCAGATGGTGAT	R: TCCAGGTCTTATCCGACTTC
IL-10	F: ATCATGACATGGACCCGGTA	R: ATTGCTCCATGACAGTTGCT
IFN-*γ*	F: CCAGATTGTTTCCCTGTACTTG	R: CATCAGAAAGGGTGTCTCTCA
TGF-*β*	F: CATCACAGAGACAGGAACCTT	R: CTTTCACATCACCACTGGAA
IgA	F: GTCACCGTCACCTGGACTACA	R: ACCGATGGTCTCCTTCACATC
IgG	F: ATCACGTCAAGGGATGCCCG	R: ACCAGGCACCTCAGTTTGG
IgM	F: GCATCAGCGTCACCGAAAGC	R: TCCGCACTCCATCCTCTTGC
*β*-Actin	F: GCACCCAGCACGATGAAAAT	R: GACAATGGAGGGTCCGGATT

**Table 3:** Differences between groups (ANOSIM).

Group	*R* value	*P* value
FBPL-FBL	0.84	0.006
FBP-FBL	0.792	0.008
FBC1-FBL	0.764	0.009
FBC1-FBPL	0.292	0.072
FBP-FBC1	0.184	0.109
FBP-FBPL	0.132	0.126

**Table 4:** Abundance of *Romboutsia* in each group.

Group	FBC1	FBL	FBP	FBPL
*Romboutsia*	62.25	3.97	61.71	67.77

## Data Availability

All the data supporting the findings of this study are adequately included within the article.
